# Penetrating Aortic Ulceration With Pseudoaneurysm and Intramural Hematoma: Emergency Department Management and Point-of-Care Ultrasound Diagnosis

**DOI:** 10.7759/cureus.27536

**Published:** 2022-07-31

**Authors:** Derrick Huang, Alexander Huttleston, Frank Fraunfelter, Leoh N Leon, Latha Ganti

**Affiliations:** 1 Emergency Medicine, HCA Florida Ocala Hospital, Ocala, USA; 2 Emergency Medicine, University of Central Florida College of Medicine, Ocala, USA; 3 Emergency Medicine, Osceola Regional Medical Center, Orlando, USA; 4 Emergency Medicine, Envision Physician Services, Plantation, USA; 5 Emergency Medicine, University of Central Florida College of Medicine, Orlando, USA

**Keywords:** peripheral arterial disease, aortic injury, emergency medicine, aortic dissection, aortic ulceration

## Abstract

Penetrating aortic ulcer (PAU) complicated by an intramural hematoma is a rare and potentially life-threatening emergency department (ED) presentation that is defined by progressive ulceration through the intima layer into the media layer of the aorta. Symptomatic PAUs can be clinically indistinguishable from other life-threatening pathologies such as aortic dissection, acute coronary syndrome (ACS), intrabdominal catastrophes as well as less lethal processes such as musculoskeletal back pain. Given the potential of PAUs to result in lethal aortic rupture and dissection, the emergency provider should maintain a high index of suspicion in patients with risk factors for aortic pathologies and utilize diagnostic modalities such as point-of-care ultrasound (POCUS) to expedite diagnosis.

## Introduction

Penetrating aortic ulcer (PAU) is a rare emergency department (ED) pathology with an estimated prevalence among patients with acute aortic syndromes (AAS) ranging from 2.3% to 7.6% [1,2]. This pathology is defined by progressive ulceration through the internal elastic lamina into the tunica media of the aorta [1]. AAS includes a family of life-threatening pathologies. These include aortic dissection (80%), followed by intramural hematoma (IMH) (15%), and PAU (5%). All are typically diseases of the geriatric population and are associated with similar underlying cardiovascular risk factors, such as atherosclerosis, hypertension, and smoking [1]. In particular, PAUs are distinctive due to an association with varied complications secondary to the progressive erosion of the aortic wall. These complications range from aortic IMH and pseudoaneurysm formation to aortic dissection and aortic rupture [1]. In the ED, the primary focus of a patient with undifferentiated abdominal pain is rapid diagnosis and treatment of life-threatening pathologies. In the geriatric population, a chief complaint of abdominal pain is complicated by a wide array of dangerous etiologies, including AAS, atypical acute coronary syndrome (ACS), gastrointestinal perforation and ischemia, and sepsis as well as limited examination findings and unreliable vital signs [3,4]. A rapid history that includes an assessment of cardiovascular risk factors and a clinical examination augmented by point-of-care ultrasound (POCUS) an be crucial in assessment [5]. Here, we present a case of PAU with pseudoaneurysm and IMH diagnosed on POCUS, requiring immediate reversal of anticoagulation and urgent surgical intervention.

## Case presentation

An 81-year-old female with a past medical history of smoking, use of apixaban for atrial fibrillation, peripheral arterial disease (PAD) with femoral endarterectomy, small bowel obstruction, and a family history of abdominal aortic aneurysm (AAA) rupture presented to the ED for diffuse abdominal pain. The pain began two days prior to arrival and had been progressively worsening. She described the pain as vague in sensation, non-radiating, and associated with nausea and dry heaving. She denied having fevers, shortness of breath, and chest pain. She reported normal bowel movements.

**Figure 1:**
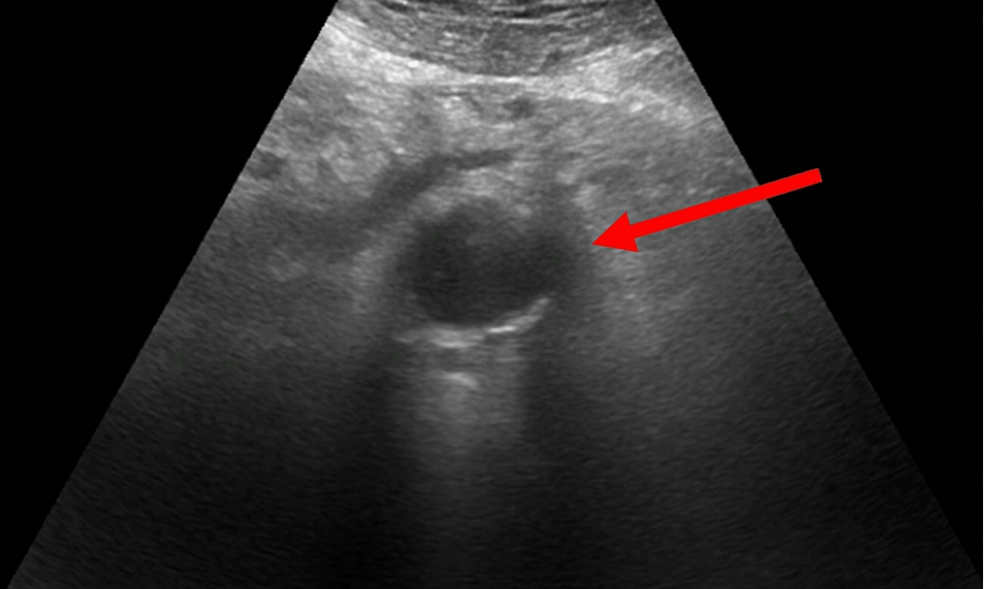
Sonogram depicting an asymmetric outpouching of the aortic wall without an intimal flap

On her initial vital signs, she had a heart rate of 110 bpm, respiratory rate of 16, blood pressure of 137/82 mmHg, and pulse oximetry of 97% on room air. She was afebrile. She was in mild distress with nonperitoneal abdominal tenderness to palpation. She had well-healed left groin and lower midline surgical incision sites. Her dorsal pedal pulse was palpable on the right and doppler positive on the left. Her initial laboratory values were significant for a hemoglobin of 11 g/dl, white blood count of 16 x 10^3^/μL, INR 1.69, and a lactic acid level of 2.4 mmol/L. Her renal function tests were unremarkable. POCUS was remarkable for an asymmetric outpouching of the aortic wall without an intimal flap (Figure 1).

**Figure 2:**
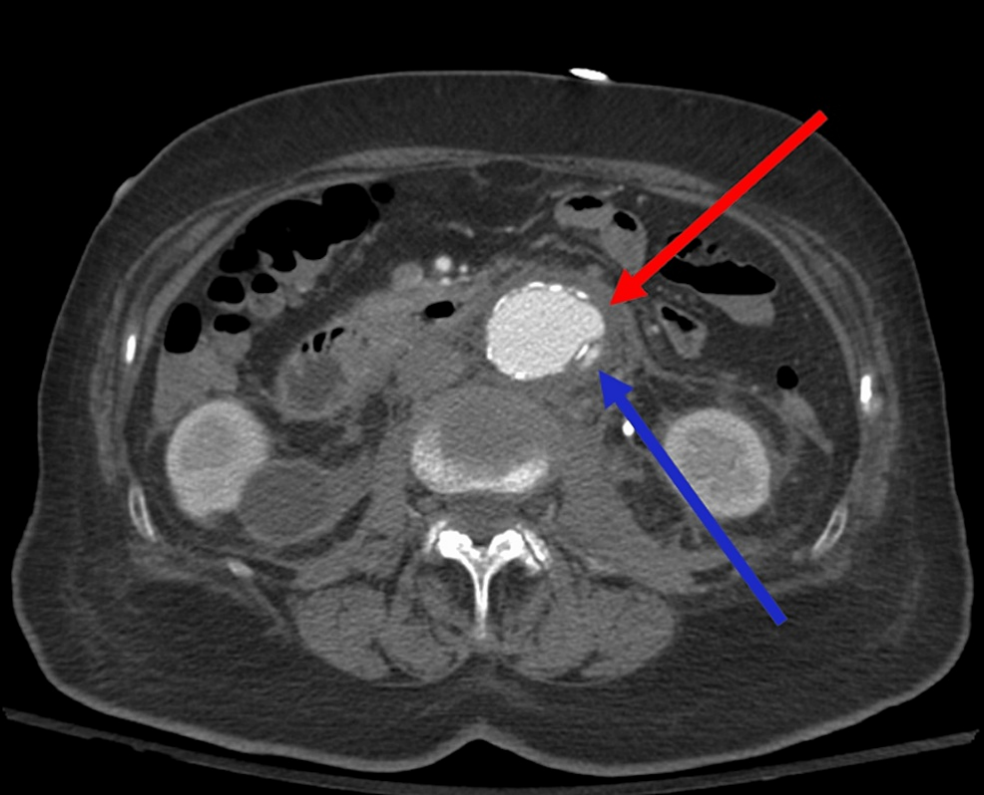
Computed tomography angiography (CTA) axial view, demonstrating aortic ulceration with pseudoaneurysm and intramural hematoma extending through the aortic wall

**Figure 3:**
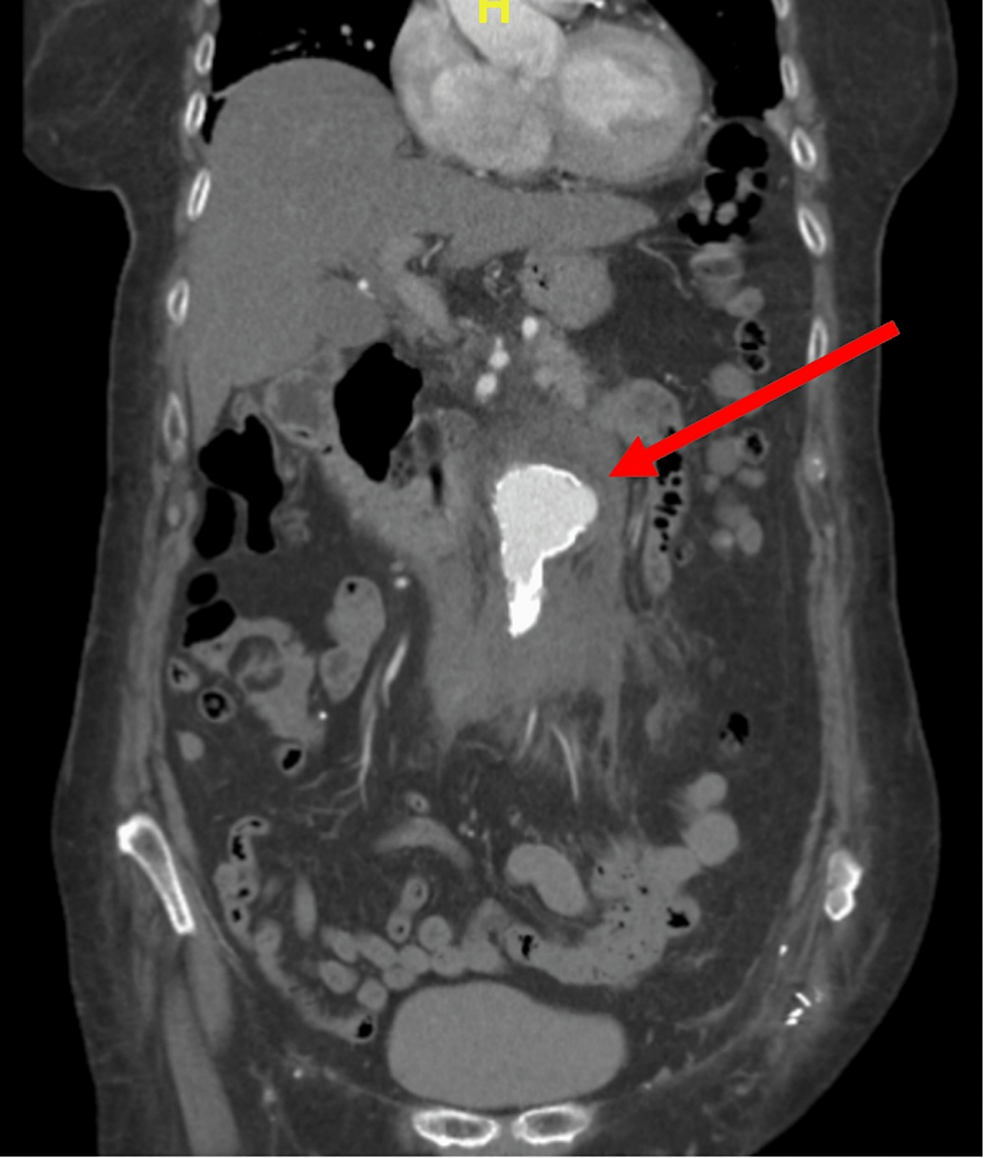
Coronal view CTA demonstrating aortic ulceration with pseudoaneurysm and intramural hematoma extending through the aortic wall

This finding prompted emergent vascular surgery consultation and use of CT angiography (CTA), which confirmed aortic ulceration with pseudoaneurysm and IMH extending through the aortic wall (Figures 2, 3).

Pain control and antihypertensive therapy with labetalol were promptly initiated, and anticoagulation was reversed with a four-factor prothrombin concentrate complex. The patient was admitted to the ICU level of care and subsequently underwent uncomplicated endovascular repair. She was ultimately discharged with an uncomplicated hospital course.

## Discussion

We report a rare case of abdominal PAU with a pseudoaneurysm and IMH in the setting of a AAA requiring reversal of anticoagulation and urgent vascular intervention. Although PAU lesions typically develop in the mid to distal descending thoracic aorta where atherosclerosis is more severe, the PAU in our patient was in the infrarenal aorta, resulting in nonspecific abdominal pain [1,6]. Our case was complicated by a dangerous and rare pathology with a nonspecific presentation of abdominal pain in a geriatric patient. Assessment of vascular risk factors and subsequent POCUS expedited assessment, timely anticoagulation reversal, and mobilization of CT imaging and vascular surgery for definitive diagnosis and intervention, respectively [5]. The family history of AAA rupture as well as age greater than 70 in our patient, in addition to the presence of vascular comorbidities such as atherosclerosis, PAD, hypertension, and smoking, all pointed to classic risk factors adjoining AAS pathologies that increase the suspicion for the aorta as the primary etiology [1]. Of note, advanced age and infrarenal AAA may be more specific risk factors for PAU relative to other members in AAS [6-8]. The presence of a symptomatic PAU with an IMH also significantly increases the risk of aortic rupture as well as disease progression, highlighting the urgency of detection in the ED and timely intervention [1,6].

PAUs can be asymptomatic and are often found incidentally on imaging [1]. Initially considered an atheromatous ulcer, PAUs are generated upon progressive ulceration beyond the intima into the media layer of the aorta [1]. When the media layer is exposed, PAUs often become symptomatic, typically manifesting with sudden, sharp pain in the back or thoracoabdomen depending on the location of the lesion, with anterior and infrascapular as well as back and abdominal pain correlating with ascending and descending localization, respectively [1]. The sympathetic nervous system is also activated in response to aortic insult, resulting in symptoms such as nausea, vomiting, tachycardia, hypertension, and diaphoresis that mandate simultaneous assessment for ACS [1]. With further ulcerative progression, many PAUs develop adventitial false aneurysms and IMHs with subsequent aortic rupture and aortic dissection [9]. There is also the potential for aortobronchial and aortoesophageal fistulation into adjacent organs with resultant hemoptysis [10]. In one longitudinal follow-up study, nearly half of PAUs progressed to aortic dissection, which can manifest with cardiac tamponade, ACS, acute aortic regurgitation, and focal neurological deficits from ischemia [11]. PAU complicated by an IMH also possesses a significant risk of rupture. In one comparative study of 198 patients, the incidence of rupture was higher compared with aortic dissection, ranging between 38% and 42% [1,8]. These ruptures may occur both early and late into the disease course, underscoring the utmost importance of rapid diagnosis and stabilization in the ED [11]. Definitive diagnosis and assessment for complications of PAUs require CTA. On CT imaging, PAUs appear as localized, crater-like, and contrast-filled outpouchings of the aortic lumen without an associated false lumen or dissection flap (Figures 2, 3) [1,2]. IMHs may be identified on CT imaging as a crescentic thickening of the aortic wall containing clotted hyperdense blood products with characteristic intramural blood pools [2].

In some centers, retrospective electrocardiographic gating has been utilized to increase the accuracy of CT in facilitating the diagnosis of more subtle PAU lesions by eliminating pulsation motion artifacts [12]. In our case, POCUS was utilized to assess for aortic pathology (Figure 1) [13]. Although the use of POCUS for PAU has not been well described in the literature, transesophageal echocardiography of the aorta is known to possess excellent accuracy in the diagnosis of aortic dissection and IMH, which may extend to PAUs as well [1,13,14]. Abdominal POCUS can also assess for aortic dissection with a sensitivity and specificity of 67%-79% and 99%-100%, respectively, while also assessing for abdominal aneurysms and peritoneal fluid with high sensitivity and specificity [15,16]. With the advantages of availability and speed inherent in ED POCUS, further studies are suggested to assess for a greater role of POCUS in the assessment of abdominal pain and diagnosis of AAS in complex patients [1].

In both symptomatic and asymptomatic cases of PAU, the primary focus in the ED is prompt initiation of anti-impulse therapy to reduce the heart rate in order to minimize the left ventricular ejection force and aortic shear stress that can worsen ulceration [1,17]. In the ED, a bolus and infusion of esmolol, a beta-adrenergic blocking agent, is the drug of choice, given its short half-life, ability to titrate to the goal heart rate of below 60 beats/minute, and suitability to initiate in patients with possible intolerance to beta blockers due to asthma or heart failure [1]. Labetalol is also an option, and diltiazem or verapamil are additional alternatives in a patient with beta blocker intolerance [17]. Systolic pressure should also be reduced to between 100 and 120 mmHg or to the lowest level feasible without malperfusion [17]. If this pressure goal is not achieved with the initial beta-blockade, a second agent can be added, typically involving intravenous nicardipine, angiotensin-converting enzyme inhibitors, nitroprusside, verapamil, or diltiazem [1,17]. Certain direct vasodilators, such as hydralazine, should be avoided due to the potential to increase aortic wall shear stress [17]. Heart rate goals, typically with beta-blockade, should be achieved first as isolated vasodilation can induce reflex sympathetic activation that enhances ventricular contraction and aortic shear stress. Adequate analgesia with opiates must also be achieved to decrease sympathetic output that can worsen tachycardia and hypertension [1].

As with a classic aortic dissection, PAUs are divided into type A and type B based on the involvement of the ascending aorta. PAUs involving the ascending aorta are type A and considered surgical emergencies given the high risk of rupture mandating operative management often with graft replacement of the ascending aorta [11]. In type B, as in our case, treatment mirrors other AASs without ascending aortic involvement with a focus on immediate medical treatment in the ED [17]. Surgical or endovascular intervention is generally indicated for refractory and severe hypertension; persistent or recurrent pain; disease progression including dissection, aneurysmal expansion, and rupture; and malperfusion with end-organ ischemia [17]. As in our case, the presence of an IMH can indicate the necessity of urgent endovascular intervention (Figures 2, 3). In PAUs with IMH, there is a significantly higher rate of disease progression to aortic rupture, hematoma expansion, or dissection with mortality rates as high as 60% when only medical therapy is utilized [9,17].

## Conclusions

PAU with pseudoaneurysm and IMH is a rare and potentially life-threatening ED pathology. Given the varied presentation and high risk of aortic rupture both early and late in the disease course, the emergency provider should maintain a high index of suspicion in patients with risk factors for aortic pathology and utilize POCUS to facilitate diagnosis and mobilization of CT imaging and the vascular team for definitive diagnosis and intervention, respectively.
